# The U.S. training institute for dissemination and implementation research in health

**DOI:** 10.1186/1748-5908-8-12

**Published:** 2013-01-24

**Authors:** Helen I Meissner, Russell E Glasgow, Cynthia A Vinson, David Chambers, Ross C Brownson, Lawrence W Green, Alice S Ammerman, Bryan J Weiner, Brian Mittman

**Affiliations:** 1 Office of Behavioral and Social Sciences Research, National Institutes of Health, Bethesda, MD, USA; 2 Division of Cancer Control and Population Sciences, National Cancer Institute, Rockville, MD, USA; 3 Division of Services and Intervention Research, National Institute of Mental Health, Bethesda, MD, USA; 4 Washington University School of Medicine, Washington University in St. Louis, St. Louis, MO, USA; 5 Department of Epidemiology & Biostatistics, School of Medicine, University of California at San Francisco, San Francisco, CA, USA; 6 Center for Health Promotion & Disease Prevention, Gillings School of Public Health, University of North Carolina at Chapel Hill, Chapel Hill, NC, USA; 7 Department of Health Policy and Management, Gillings School of Public Health, University of North Carolina at Chapel Hill, Chapel Hill, NC, USA; 8 VA Greater Los Angeles Healthcare System, Sepulveda, CA, USA

**Keywords:** Training, Dissemination, Implementation

## Abstract

**Background:**

The science of dissemination and implementation (D&I) is advancing the knowledge base for how best to integrate evidence-based interventions within clinical and community settings and how to recast the nature or conduct of the research itself to make it more relevant and actionable in those settings. While the field is growing, there are only a few training programs for D&I research; this is an important avenue to help build the field’s capacity. To improve the United States’ capacity for D&I research, the National Institutes of Health and Veterans Health Administration collaborated to develop a five-day training institute for postdoctoral level applicants aspiring to advance this science.

**Methods:**

We describe the background, goals, structure, curriculum, application process, trainee evaluation, and future plans for the Training in Dissemination and Implementation Research in Health (TIDIRH).

**Results:**

The TIDIRH used a five-day residential immersion to maximize opportunities for trainees and faculty to interact. The train-the-trainer-like approach was intended to equip participants with materials that they could readily take back to their home institutions to increase interest and further investment in D&I. The TIDIRH curriculum included a balance of structured large group discussions and interactive small group sessions.

Thirty-five of 266 applicants for the first annual training institute were accepted from a variety of disciplines, including psychology (12 trainees); medicine (6 trainees); epidemiology (5 trainees); health behavior/health education (4 trainees); and 1 trainee each from education & human development, health policy and management, health services research, public health studies, public policy and social work, with a maximum of two individuals from any one institution. The institute was rated as very helpful by attendees, and by six months after the institute, a follow-up survey (97% return rate) revealed that 72% had initiated a new grant proposal in D&I research; 28% had received funding, and 77% had used skills from TIDIRH to influence their peers from different disciplines about D&I research through building local research networks, organizing formal presentations and symposia, teaching and by leading interdisciplinary teams to conduct D&I research.

**Conclusions:**

The initial TIDIRH training was judged successful by trainee evaluation at the conclusion of the week’s training and six-month follow-up, and plans are to continue and possibly expand the TIDIRH in coming years. Strengths are seen as the residential format, quality of the faculty and their flexibility in adjusting content to meet trainee needs, and the highlighting of concrete D&I examples by the local host institution, which rotates annually. Lessons learned and plans for future TIDIRH trainings are summarized.

## Background

One of the most critical issues impeding improvements in public health today is the enormous gap between what we know can optimize population health and healthcare delivery and what actually gets implemented in everyday practice [1]. The science of dissemination and implementation (D&I) seeks to address this gap by understanding how to create, evaluate, report, disseminate, and integrate evidence-based strategies to improve health and prevent disease in clinical and public health practice settings [2]. Academic and government institutions have been focusing on D&I in recent years [3-6]. Since 2000, the National Institutes of Health (NIH) have been advancing the science of D&I through a variety of initiatives, including research funding opportunities, conferences, and workshops (http://www2.niddk.nih.gov/NR/rdonlyres/E767D678-B5B3-426E-B954-711510ACF886/0/confpublication.pdf, http://obssr.od.nih.gov/scientific_areas/translation/dissemination_and_implementation/index.aspx) [7]. These efforts have been successful in establishing D&I research as a legitimate field of scientific inquiry and have stimulated growth in the number of D&I grant applications submitted and funded (interested readers can search trends in NIH-funded D&I research at http://www.report.nih.gov). However, for emerging fields of science to flourish, a complementary investment in education and training is necessary to facilitate the establishment of a cadre of investigators to move the field forward, to serve as peer reviewers for the growing number of grant applications and journal submissions of manuscripts, and to fill the growing number of faculty positions dedicated to D&I [8,9].

D&I research draws from a variety of clinical, public health, behavioral, and social science disciplines and employs approaches and methods that in the past have not been taught comprehensively, if at all, in most graduate degree programs. Perhaps because it is inherently interdisciplinary, with no obvious departmental home, few university-based training opportunities exist in the United States for investigators interested in pursuing D&I research (see http://obssr.od.nih.gov/scientific_areas/translation/dissemination_and_implementation/DI2012/resources/ImplSci-TrainingDirectory-Feb2012.pdf for 2012 directory of implementation science training programs compiled by the VA Center for Implementation Practice and Research Support). A content analysis of the training programs of the 60 Clinical and Translational Science Awards (CTSAs) in the United States found only three CTSAs with significant offerings in training for D&I research (Wes Gibbert, unpublished). The University of California, San Francisco, offers an Implementation Sciences track within a 36-quarter unit Master’s program in Clinical Research (http://www.epibiostat.ucsf.edu/courses/masters.html#tracks), and a 6-quarter unit Certificate Program in Implementation and Dissemination Science (http://www.epibiostat.ucsf.edu/courses/implementation_research.html) [3]. Tufts University’s Clinical and Translational Science Graduate Program offers a concentration in Evidence-Based Clinical Effectiveness Research at both the Master of Science and the PhD levels (http://www.tuftsctsi.org/Education-and-Career-Development/Clinical-and-Translational-Science-Graduate-Program.aspx?c=129944762486592470). The University of Iowa offers a one-year Certificate in Translational and Clinical Investigation ( http://icts.uiowa.edu/content/certificate-translational-and-clinical-investigation) based on a 2010 White Paper from the CTSA Comparative Effectiveness Research Workgroup on Workforce Development and the above Tufts curriculum.

A summer training program for investigators new to D&I, the Implementation Research Institute (IRI), was established at Washington University in St. Louis with support from a grant from the National Institute of Mental Health. This two-year training in implementation science is specifically for mental health services researchers (http://cmhsr.wustl.edu/Training/IRI/Pages/ImplementationResearchTraining.aspx). IRI Fellows attend a one-week training each year, where they receive individualized mentoring and visit active D&I research study sites to gain real-world perspective on the complexity of conducting this type of research.

The U.S. Department of Veteran Affairs offers Enhancing Implementation Science in VA (EIS) to investigators interested in developing their implementation research knowledge and skill. This program, attended by VA and non-VA researchers, was delivered as in-person, two-day training meetings in 2010 and 2011, while the 2012 program was offered via Cyber Seminar (http://www.queri.research.va.gov/meetings/eis/). At a national level, the Canadian Institutes of Health Research (CIHR) has developed a training initiative to address the shortage of trained investigators in the science and practice of knowledge translation (KT). Their program includes modular courses, a national seminar series, an annual Knowledge Translation Summer Institute, and yearly research meetings [4,5]. Clearly, the few specialized D&I training opportunities currently available are not adequate for meeting the need to prepare public health and clinical researchers in the science of D&I.

To address the dearth of training opportunities in D&I, the NIH Office of Behavioral and Social Sciences Research, in collaboration with the National Cancer Institute, the National Institute of Mental Health, and the US Department of Veterans Affairs developed a five-day Training Institute for Dissemination and Implementation Research in Health (TIDIRH) to be conducted on an annual basis at institutions around the country on a rotating basis. The purpose of this paper is to describe the goals and process of developing TIDIRH, present short-term evaluation results from the first training session held in August 2011, and describe future directions and resources for building capacity for D&I research.

## Methods

### Working definitions

Multiple and often inconsistent definitions of D&I research exist. Lack of consensus surrounding terminology is not uncommon when fields of study are new—especially when key concepts have roots in a variety of disciplines [6,10]. Acknowledging this diversity in definitions, we adopted the following for purposes of TIDIRH.

*Dissemination research* is the systematic study of processes and factors that lead to widespread use of an evidence-based intervention by the target population. Its focus is to identify the best methods that enhance the uptake and utilization of the intervention [10].

*Implementation research* seeks to understand the processes and factors that are associated with successful integration of evidence-based interventions within a particular setting (*e*.*g*., a worksite or school). Implementation research assesses whether the core components of the original intervention were faithfully transported to the real-world setting (*i*.*e*., the degree of fidelity of the disseminated and implemented intervention with the original study) and is also concerned with the adaptation of the implemented intervention to the local context [10].

### Goals

The goal of TIDIRH is not only to prepare investigators to conduct research that addresses the complex process of integrating research into policy and practice but also to cultivate interest for D&I research at institutions around the country. Consequently, in addition to conducting an intensive annual five-day residential training institute, TIDIRH will rotate host institutions annually in order to engage local faculty with interest in building D&I programs. As shown below, TIDIRH faculty and guest lecturers in 2011 consisted of leading experts (researchers, practitioners, and teachers) in theory; implementation and evaluation approaches to D&I; creating partnerships and multilevel transdisciplinary research teams; research design, methods, and analyses appropriate for D&I investigations and conducting research at different and multiple levels of intervention (*e*.*g*., clinical, organizational, community, policy). To provide an international perspective, TIDIRH also included faculty from the World Health Organization, as well as other faculty who conduct international research. While there will be continuity of core faculty from year to year, a 40–60% turnover in faculty is expected to capitalize on local talent and highlight examples from host institutions with interest in D&I. The primary objective of the TIDIRH is to increase the submission rate and quality of D&I grant applications and publications by trainees. A second objective is to use a “train-the-trainer-like model,” in which program participants are expected to return to their home institutions and share what they have learned at the institute (*e*.*g*., giving talks, leading seminars, forming new collaborations, mentoring, and submitting D&I grant proposals as part of transdisciplinary research teams), to further build a network of capacity for conducting D&I research.

2011 TIDIRH Faculty

Core Planning Faculty

Alice S. Ammerman, DrPH, RD, University of North Carolina at Chapel Hill

Ross C. Brownson, PhD, Washington University in St. Louis

David Chambers, DPhil, National Institute for Mental Health

Russell E. Glasgow, PhD, National Cancer Institute

Lawrence W. Green, DrPH, DSc (Hon), University of California, San Francisco

Sarah Johnson, PhD, National Institutes of Health

Helen I. Meissner, ScM, PhD, National Institutes of Health

Brian Mittman, PhD, VA Greater Los Angeles Healthcare System

Kurt C. Stange, MD, PhD, Case Western Reserve University

Cynthia A. Vinson, MPA, National Cancer Institute

Maihan B. Vu, DrPh, MPH, University of North Carolina at Chapel Hill

Bryan J. Weiner, PhD, University of North Carolina at Chapel Hill

Guest Faculty

Gary G. Bennett, PhD, Duke University

C. Hendricks Brown, PhD, MA, University of Miami

Marci K. Campbell, PhD, University of North Carolina at Chapel Hill

Timothy Carey, MD, MPH, University of North Carolina at Chapel Hill

Lynn Etheredge, George Washington University

Kristen Hasmiller Lich, MHSA, PhD, University of North Carolina at Chapel Hill

Jennifer Leeman, MDiv, DrPH, University of North Carolina at Chapel Hill

Shawna L. Mercer, MSc, PhD, Centers for Disease Control and Prevention

Joseph P. Morrissey, PhD, University of North Carolina at Chapel Hill

Enola K. Proctor, Washington University in St. Louis

Carmen D. Samuel-Hodge, PhD, MS, RD, University of North Carolina at Chapel Hill

Nhan T. Tran, MHS, PhD, World Health Organization

Robin Yabroff, PhD, MBA, National Cancer Institute

Elizabeth M. Yano, PhD, MSPH, VA Greater Los Angeles, UCLA School of Public Health

### Structure

The Prevention Research Center at the University of North Carolina, Chapel Hill, was selected in 2011 to serve as the first TIDIRH host. Twelve core faculty, which included five NIH staff, one VA staff, leading experts in D&I research, and three host faculty, were charged with planning the curriculum and agenda. To the extent possible, the approach to TIDIRH was to structure a curriculum that would be interactive and flexible. Core faculty participated in the seven-month planning process and was expected to attend the full five-day course. Guest faculty were asked to attend TIDIRH at least one day prior to their lecture, if possible, to allow opportunity to interact with trainees and become acquainted with the TIDIRH format.

Consistent with the train-the-trainer-like goal of TIDIRH, faculty was given specific guidance for preparing talks and a detailed presentation template. The intent was that trainees should be equipped with materials that they could readily adapt to give similar lectures at their home institutions, networks, or professional conferences following TIDIRH. While it is unrealistic to expect trainees to become expert in the science of D&I with only a five-day course, we did think it reasonable for them to share knowledge gained with their colleagues and to convey the importance of pursuing D&I as an important and legitimate area of scientific inquiry. Consequently, faculty was required to include detailed notes below each slide with comments so that others could deliver the presentations. They also were asked to include a final slide of take-home points and up to three key references. To facilitate interaction, lecturers were asked to include thought or discussion questions in their presentations and to plan to devote a minimum one-third of the session time for discussion. All slides were reviewed by core faculty prior to the institute and feedback was provided. All PowerPoint slides and references were given to trainees for future use, and all lectures are in the public domain and posted on the TIDIRH website (http://conferences.thehillgroup.com/OBSSRinstitutes/TIDIRH2011/index.html).

Core faculty met weekly to plan the TIDIRH curriculum and develop a working model to guide the training. Ward’s [11] conceptual framework of translating knowledge into action was adapted for use in TIDIRH. The idea of the framework was to emphasize that clinical, community, and public health practice needs evidence that is rigorous, relevant to fit local context, and adapted as necessary, balancing fidelity to the evidence-based principles and customization with engagement of local stakeholders [12,13].

### Curriculum

As shown below, the curriculum included a balance of structured content topic areas and interactive small group and personalized sessions on individual trainee projects. The week was designed so that core material covered each day could be used subsequently in developing sections of trainee research proposals (discussed during the small group session each afternoon). As can be seen, content included both substantive (*e*.*g*., designing for dissemination, balancing fidelity and adaptation, global health, participatory approaches, policy applications) and methodological issues (*e*.*g*., research design, measurement and evaluation, rapid learning, pragmatic trials, comparative effectiveness research, system dynamic tools). A moderate amount of real-time adjustment and schedule/topic adjustment was done throughout the week based upon daily experiences and the prioritization of sessions by the trainees. For example, case study sessions originally on the agenda for days 2, 3, and 4 were cancelled in order to allow more time for discussion sessions with the group as a whole, as requested by trainees. The informal interactions and chance to interact with faculty and other trainees individually and in groups were also an important part of the design of the training experience and enhanced by faculty and trainees sharing all meals in the same dining room, with faculty rotating among trainees at different tables.

Training Institute for Dissemination and Implementation Research in Health 2011 Curriculum

Day 1: Introduction, Overview, and Design

1. Participant Introductions

2. Working Model for the Week

3. Dissemination Science

4. Implementation Science: An Organizational Perspective

5. Designing for Dissemination

6. Designs and Types of Evidence for D&I Research

7. D&I Funding Opportunities

8. Breakout groups to discuss individual projects 1: Specific Aims/Theory

Day 2: Design and Measurement

1. Balancing Fidelity and Adaptation: If We Want More Evidence-Based Practice, We Need More Practice-Based Evidence.

2. Concurrent Session: Realist Synthesis: Building the Evidence Base for D&I Research

3. Concurrent Session: In Search of Synergy: Strategies for Combining

4. Interventions at Multiple Levels

5. Design, Measurement, and Evaluation: Part 1

6. Design, Measurement, and Evaluation: Part 2

7. Economic Studies in D&I Research

8. Breakout groups to discuss individual projects 2: Design and Measurement

Day 3: Intervention and Methods

1. Global Applications of Implementation Research

2. A Rapid-Learning Healthcare System: Using *in silico* Research, Adopting “Best Practices”

3. Participatory Approaches: How Can CBPR Guide Translation and Dissemination?

4. Intensive Behavioral Weight Loss in Public Health Settings

5. Breakout groups to discuss individual projects 3: Methods

6. Concurrent Session: Comparative Effectiveness Research: Moving the Field Ahead and Disseminating Results

7. Concurrent Session: System Dynamics Tools for D&I

8. Concurrent Session: eHealth and D&I Research: What Next?

Day 4: Scale-up

1. Scale-Up and Spread

2. Sustainability

3. Grant Review Skit

4. Funding Q&A

5. Clinical D&I Research: A Line of Inquiry and Spin-offs

6. Research Exemplars and Discussion: *Tobacco*

7. Policy Dissemination Research

8. Breakout groups to discuss individual projects 4: Scale-up, Grant Application Issues (Funding mechanisms, Sources, Program Officers, etc.)

Day 5: Evidence

1. External Validity: Why it Matters

2. Practice-Based Research Networks: Participatory Laboratories for Discovery, D&I

3. Evidence-Based Recommendations: Context and Opportunities

4. Pragmatic Trials 101

5. Train the Trainer & Post-Course Expectations

### Application process

TIDIRH’s call for applications was disseminated through the Office of Behavioral and Social Sciences Research and other NIH listservs containing potentially interested subscribers and announced at the 2011 NIH Conference on the Science of Dissemination and Implementation. Applications were open to both new and experienced investigators. However, to be eligible, participants could NOT have received major research funding, defined as R18, R01, or R01-equivalent funding for D&I research. Preference was given to applicants who demonstrated experience with, or potential for, working effectively in transdisciplinary teams and who had strong partnerships with—or were embedded within—healthcare delivery, public health, or community-based organizational settings. In addition, to be eligible, participants had to meet all of the following criteria:

• Hold a doctoral level degree (PhD, ScD, MD, DrPH, DO, DVM, DNSc, etc.)

• Have demonstrated experience and expertise in health science (*e*.*g*., medicine, behavioral medicine, nursing, medical anthropology, health economics, health policy)

• Have a D&I research concept to bring to the institute and develop throughout the training

• Be able to attend the entire residential training five-day institute, if accepted

• Provide a letter of support from their organization that there was institutional commitment to D&I research and that this training would benefit the organization.

## Results

Two-hundred sixty-six investigators applied in the first year through a competitive process to fill 30 NIH and 5 VA available openings. The size of the class was designed to be small enough to maintain an interactive environment and to enable full support of trainees given budgetary limits. Applicants were required to submit the following:

• A one-page statement indicating the basis for interest in attending the course and plans to use the knowledge gained to promote the science of D&I at the home institution

• A letter from employer/institution supporting applicant time away to attend the course

• A current curriculum vitae (CV)

• A 1.5- to 2-page concept paper describing the D&I research project the applicant planned to develop during the training. This paper had to include the specific aims and general approach. In addition, applicants had to describe (a) the evidence-based practice or innovation to be implemented (or studied within the context of a natural experiment of implementation or adaptation) or disseminated, (b) the type of implementation strategy or approach envisioned (if planning an interventional study), and (c) the type of evaluation or research plan to be employed.

Two independent reviewers (from a pool of 47 reviewers who were established D&I investigators and NIH staff) rated each application. Applicants were rated on the following criteria:

1. Overall quality and quantity of scholarship and research relevant to stage in career

2. Capacity (and/or potential) for working effectively in transdisciplinary teams

3. Interest in and commitment to future transdisciplinary research in D&I

4. Evidence of D&I research support, collaboration potential, institutional support, and access to mentors and colleagues

5. Likelihood that TIDIRH training would lead to a successful D&I application for NIH or VA or comparable R01 funding

6. Quality and potential impact of D&I research proposal submitted

Trainees were ranked by review score. In order to maximize geographic diversity, no more than two applicants could be accepted from the same institution. Final selections were based on rank order and diversity, with the top 30 non-VA applicants to be supported by NIH and the top 5 VA applicants to be supported by the VA. Of the 35 applicants accepted, 27 had PhDs, 7 had MDs, and one was a DDS. Two applicants were unable to attend TIDIRH, resulting in a total of 33 who completed the five-day training. Their doctoral training included the following disciplines: psychology (12 trainees), medicine (6 trainees), epidemiology (5 trainees), health behavior/health education (4 trainees), and 1 trainee each from education & human development, health policy and management, health services research, public health studies, public policy, and social work. Trainees were diverse with respect to the areas in which they wanted to focus their D&I studies, ranging from implementation of health promotion interventions (*e*.*g*., weight management, cancer screening), mental health treatment, hospice, and quality improvement for hospitals to tobacco policy. Accepted applicants were asked to complete a needs assessment prior to the training, for faculty to gain understanding of participant’s interests and level of expertise in D&I and to help tailor the training and curriculum. Less than five applicants ranked themselves as “expert” on a given area of D&I knowledge/experience (*e*.*g*., design, methods and measurement, approaches to scale-up in D&I research), while 4–17 applicants self-rated as “competent” on a given D&I topic.

### TIDIRH evaluation and follow-up

Concurrent evaluations regarding session relevance and quality of instruction concluded each day’s meeting with trainees. Additionally, core faculty met at the end of each day to adjust session content and schedule in order to address trainee feedback and adjust and finalize the next day’s schedule and format to better meet trainee needs and emerging issues. Overall, at the end of the five-day training, an overwhelming majority of trainees agreed/strongly agreed on the final evaluation that the agenda was relevant to their needs/interests (97%), that the teaching strategies were appropriate (97%), and that they were confident in their ability to apply the skills and knowledge gained throughout the week (88%). One trainee wrote, “The success to this week is a real testament to the leaders in this field, all of the attention paid to mixing inspirational with practical advice on building the field and adapting the schedule to address these issues paid off.”

Trainees suggested that the area where TIDIRH could most improve was to provide more training in assembling and leading a multidisciplinary team and assistance with study design. Additional suggestions included more one-on-one mentorship and more time to work on individual proposals. Of the 16 trainees who responded to the question, 14 (88%) said that they would like to be considered as a guest lecturer at a future TIDIRH.

At the conclusion of the week’s training, participants were queried on ways faculty and the cohort of trainees could continue to support their efforts to conduct D&I research. Most trainees thought that an interactive web-networking platform would be helpful to maintain and maximize their D&I research connections. Consequently, a platform to provide a forum for trainees to get feedback on grant applications, share lectures and papers, post training and funding opportunities, and communicate in general about news and issues related to the field was created on NING (http://tidirh2011.ning.com).

A follow-up evaluation was conducted six months posttraining to assess the extent to which TIDIRH was meeting its goal to prepare investigators to conduct D&I research. Thirty-two out of 33 trainees (97%) completed and returned the evaluation. Table 1 shows that TIDIRH participants have used their training not only to submit D&I grant applications but also to share their knowledge about D&I with others. A majority (72%) of TIDIRH participants initiated a new D&I grant application following the training; 28% were successful in obtaining D&I grant support by the six-month assessment. Almost all (97%) trainees used the knowledge gained from TIDIRH to influence thinking of colleagues about D&I science, including informal and formal meetings with colleagues, networks and listservs, formal presentations at home institutions and scientific conferences, teaching and advising students, and leading teams to collaborate on D&I research projects. Only about a third of trainees had been using the online networking platform, but 67% participated in a conference call that was held to discuss a specific D&I funding opportunity. A majority (62%) of trainees have had professional contact with faculty or other trainees since TIDIRH. Results suggested that trainees would find additional posttraining useful, such as expert lectures (81%), attending additional training programs (74%), and dedicated sessions at national conferences (61%).

**Table 1:** **TIDIRH 2011 trainee six-month follow-up evaluation**^**a**^

	**n**	**%**
**TIDIRH contribution to grants**		
Initiated a new D&I grant application	23	72
Revised a new D&I grant application	5	16
Modified a previously non-D&I grant application to include D&I components	6	19
Had a D&I grant application funded	9	28
		
**Ways used knowledge and skills gained from TIDIRH**		
Used in conducting ongoing implementation studies or how to approach D&I science and related research	20	65
Used to influence the thinking of others about D&I science through informal conversations with colleagues	30	97
Used to influence thinking of others about D&I science through interactions with interdisciplinary research groups (*e*.*g*., CTSA, research networks)	24	77
Used to engage with community groups or stakeholders to plan projects	20	65
Have not used yet but plan to do so soon	3	13
Have not used and don’t have current plans to do so	0	0
		
**Participation in post-TIDIRH activities**		
Online networking platform	7	33
Conference call to discuss funding opportunity	14	67
		
**Professional contact with any faculty or trainees since TIDIRH**		
Yes	20	62
		
**Other forms of post-TIDIRH activities that would help**		
Follow-up lectures (conference call or webinar) by TIDIRH faculty or other experts	25	81
Follow-up informal Q&A discussion sessions (conference call or webinar) with TIDIRH faculty	16	52
Additional opportunities for interaction with fellow TIDIRH trainees (without faculty)	13	42
Local mentoring	15	48
Attendance at additional training programs	23	74
Dedicated session(s) at 2012 NIH D&I conference	19	61

^a^Includes 32 of the 33 (97%) trainees who responded to evaluation.

TIDIRH = Training Institute for Dissemination and Implementation Research in Health; D&I = dissemination and implementation; CTSA = Clinical and Translational Science Awards; NIH = National Institutes of Health.

## Conclusions and discussion

A comprehensive approach to building capacity for D&I research necessitates investment in training new investigators to grow the field. TIDIRH was developed to address a lack of D&I training opportunities available to investigators in the United States and elsewhere. An intended feature of TIDIRH was to offer a transdisciplinary program of training that was not restricted to a focus on a particular disease or implementation setting (*e*.*g*., clinic, community). TIDIRH offered a unique opportunity to train a diverse group of investigators in how best to effectively integrate research evidence into clinical and public health practice and policy. By all accounts, we judge the inaugural session of TIDIRH a success. Trainees not only reported using skills gained from their TIDIRH experience to secure funding for D&I research, they also are helping to build an appreciation for and understanding of D&I as an important scientific pursuit through formal and informal interactions with their colleagues.

Trainees provided feedback throughout the week on how to improve TIDIRH. Even though the agenda built in time for discussion during and after sessions, we still had more content than could be fit into the long days. We learned that we must build in enough flexibility during the training to make iterative corrections for time and content. It is likely that we will continue to struggle with achieving the “right” balance of covering what we believe to be essential material, while allowing enough time for questions, interaction, emerging issues, and development of individual projects. One approach we are taking for TIDIRH 2012 is to assign and send readings for most of the presentations to trainees four to six weeks prior to the training. The recent publication of the first comprehensive text on D&I research in health [2] will be used as a text and help to provide background to trainees prior to meeting.

Fine-tuning the TIDIRH curriculum will continue to be a dynamic process. In addition to turnover of faculty from year to year (resulting from the change in host institution), maturing of the field and changes in healthcare delivery and policy will likely necessitate modifications to the TIDIRH curriculum. Consequently, we will continue to engage a group of core planning faculty at least six to seven months prior to the training, in order to allow for appropriate changes to TIDIRH content. For example, plans for the coming year include presentations from officials from the new Patient Centered Outcomes Research Institute (http://www.pcori.org).

While developing the TIDIRH curriculum, core faculty devoted much time to agreeing on a single ‘model’ to guide the training. In retrospect, however, we realize that it is not necessary, practical, or even advisable to agree on a single model for TIDIRH. Feedback from trainees suggests that they would have found it more useful to focus on a rationale for why D&I research is needed than to have consensus on a model to guide the field. Trainees would rather have available a presentation defining and justifying D&I research to use when presenting to their colleagues, especially colleagues in the basic biomedical sciences who often are unfamiliar with this type of applied research. For 2012, the training will begin with such a lecture and will include emphasis on commonalities among the over 60 models, theories, and frameworks that have been published for D&I [14]. Multiple models will be discussed throughout the week to guide different aspects of the curriculum and enable trainees to better select and use models to inform their D&I research.

One of the strengths of TIDIRH is that it was open to trainees representing diverse backgrounds, experiences, and interests and that it was not institution bound. However, this strength also presented a challenge. TIDIRH trainees were interested in developing a range of D&I studies in a variety of VA, clinical, community, and policy settings. Our interdisciplinary approach, however, meant that tailoring the curriculum to separate disciplines, disease outcomes, or contexts of interest to trainees was not possible. The small group sessions, which were supposed to provide an opportunity for trainees to get feedback on their individual projects, did not fully satisfy the desire for more tailored area-specific instruction. Consequently, in planning future institutes, we will try to organize trainees with like interests/implementation settings for the small group sessions and also plan to build in more one-on-one time with expert faculty to provide feedback on individual projects.

A related challenge to meeting the diverse interests of trainees was that TIDIRH was open to both junior and more established investigators. Training junior investigators, of course, has the advantage of developing new cohorts of investigators who may devote their careers (both research and teaching) to D&I. However, conducting D&I research requires a different skill set than basic scientists or even applied intervention researchers possess, and we believed that more experienced investigators interested in changing their focus to D&I would benefit from the training. We also reasoned that more established investigators would provide the added advantage of being better positioned to build capacity for D&I research at their home institutions and professional organizations. Nevertheless, we struggled with assessing the level at which an investigator was “too senior” to gain much from the training and the extent to which giving a valuable spot at TIDIRH to an already established investigator was justified. While we did not entirely resolve this dilemma, for TIDIRH 2012, we decided to give preference to applicants who demonstrate experience with, or within, healthcare delivery or community-based networks to facilitate more rapid and broader translation of TIDIRH training.

TIDIRH will continue its plan to increase capacity for D&I research by rotating local host institutions each year and by using a train-the-trainer approach in its design. Yet this annual summer institute, in addition to the other offerings currently available, is unlikely to meet the demand for comprehensive D&I training in the United States. Over 200 applications to TIDIRH were received in 2011 and again in 2012. Although the short-term evaluation presented here suggests that TIDIRH is beginning to meet its goals, there continues to be a need for sustainable strategies that have wider reach and at the same time build capacity for D&I training at academic and related health research institutions. In addition to the training opportunities mentioned earlier in this paper, resources such as the *Dissemination & Implementation in Health* e-Newsletter, which provides monthly updates on research practice and policy activities in D&I (email wynne.norton@gmail.com to subscribe) and websites that are designed to increase the D&I potential of effective interventions and affiliated products (*e*.*g*., Make Research Matter (http://www.makeresearchmatter.org/ and Cancer Control P.L.A.N.E.T (http://cancercontrolplanet.cancer.gov/) help to create a community of practice. The NIH and VA will continue to support the annual TIDIRH but also are pursuing additional avenues for building the field, such as web-based learning opportunities. Continued investment in training the next generation of scientists is essential not only to advance the field of D&I but ultimately is needed to ensure that advances in science make a difference in improving health.

## Competing interests

Dr. Brian Mittman is Editor-in-Chief Emeritus of *Implementation Science*. Drs. Russell E Glasgow and Lawrence W Green are members of the Senior Advisory Board of *Implementation Science*. Dr. Bryan J Weiner is a member of the Editorial Board of *Implementation Science*.

## Authors’ contributions

HIM wrote manuscript. REG wrote abstract & provided input and edits. CAV abstracted data and provided input and edits, DC provided input and edits, RCB provided input and edits, LWG provided input and edits, ASA provided input and edits, BJW provided input. BM provided input. All authors read and approved the final manuscript.
